# An ecological study regarding the association between paraquat exposure and end stage renal disease

**DOI:** 10.1186/s12940-022-00946-9

**Published:** 2022-12-12

**Authors:** Gerald McGwin, Russell L. Griffin

**Affiliations:** Department of Epidemiology, School of Public Health, University of Alabama at Birmingham, 700 South 18th Street, Suite 609, Birmingham, AL 35294-0009 USA

**Keywords:** Herbicide, Renal, Paraquat, ESRD

## Abstract

**Background:**

Persons who experience paraquat poisoning rapidly develop damage to a variety of organ systems including acute kidney injury (AKI), the occurrence of which is associated with an increased risk of death. However, little is known about the effects of chronic paraquat exposure on renal function and the onset of chronic renal disease. The objective of the current study is to assess the association between paraquat exposure and the incidence of end stage renal disease (ESRD) in the United States.

**Methods:**

Data on the incidence of ESRD for the period 2010 through 2017 and kilograms of paraquat use per square mile for each county in the conterminous United States was obtained from the United States Renal Data System (USRDS) and the National Water Quality Assessment (NAWQA) Program, respectively. Negative binomial regression was used to estimate rate ratios (RRs) and 95% confidence intervals (CIs) for the association between quartiles of paraquat exposure and the incidence of ESRD.

**Results:**

The incidence of ESRD increased with increasing paraquat density. Based on a 20-year exposure lag, those in the highest paraquat density quartile had a 21% higher rate of ESRD compared to the lowest quartile whereas for a 15-year lag the increase was 26%. Adjusted associations were attenuated though still followed an increasing linear trend across quintiles.

**Conclusions:**

The results of this study are consistent with a large number of studies documenting a high incidence of AKI and a small number of studies chronic renal disease following acute and chronic paraquat exposure, respectively. While the pathophysiological mechanisms underlying kidney injury following paraquat poisoning are well understood, more research is necessary to understand the natural history of chronic kidney disease due to chronic paraquat exposure.

**Supplementary Information:**

The online version contains supplementary material available at 10.1186/s12940-022-00946-9.

## Background

The herbicide paraquat (1,1′-dimethyl-4,4′-bipyridinium dichloride) was first marketed in 1962 for the control of annual grasses, broad leaf weeds, and the tips of established perennial weeds [1]. In the United States (US), the Environmental Protection Agency (EPA) has classified paraquat as “restricted use” and, as such, can only be used by licensed pesticide applicators [2]. The US Geological Survey estimated that in 2017, 10 million pounds of paraquat active ingredient was applied [3]. Paraquat dichloride is soluble in water, has been shown to be immobile in soil, is resistant to microbial degradation, and does not hydrolyze or photodegrade in aqueous solution [4]. Non-occupational human exposures may occur through food and drinking water from application to growing crops, and thereafter reaching surface and ground water [5]; furthermore, aerial application is a concern due to spray drift [6]. Acute paraquat intoxication results in a range of health effects including acute kidney injury (AKI) and multiple organ failure; the mortality rate is exceedingly high [7]. Regarding chronic exposure, Parkinson’s disease has been the most commonly investigated adverse health effect with most studies reporting a positive association [8–18]; however, lung function and respiratory effects [19–24], cancer [25], diabetes [26], myocardial infarction [27], among other conditions have been investigated. The adverse effects of acute paraquat exposure on kidney function have been known since the herbicide was first marketed in the 1960s. Since that time there have been a large number of reports detailing the clinicopathologic features of paraquat associated acute kidney injury [28–30], the mechanism by which this damage occurs has also been widely investigated [31, 32]. In contrast, there has been little research regarding the impact of chronic paraquat exposure on renal disease [33–36]. The objective of this study is to investigate the association between county-level measures of paraquat use and the incidence of ESRD.

## Methods

### Study design

The current study uses an ecological study design to compare the county-level incidence of ESRD and paraquat exposure for the period 2010 through 2017 in the conterminous United States.

### Data sources

The current study relied on data from the United States Renal Data System (USRDS). The National Institute of Diabetes and Digestive and Kidney Diseases (NIDDK) funds the USRDS, and data originates from the Centers for Medicare and Medicaid Services (CMS), the United Network for Organ Sharing (UNOS), and the ESRD networks. The USRDS database includes information on all ESRD patients in the United States, regardless of insurance coverage and age. The USRDS is a surveillance system and defines ESRD based on treatment (i.e., incident ESRD cases are patients starting any modality of dialysis or transplantation and identified by medical providers and institutions) [37]. For the purposes of the current study, the USRDS’s Data Extraction System for Kidney Related Information & Basis Epidemiology (DESKRIBE) was used to enumerate county level counts of all adult (aged 18 years and older) incident ESRD patients between 2010 and 2017 [38].

The National Water Quality Assessment (NAWQA) Program was established in 1991 to address the status of water quality in the United States. Among the activities of the NAWQA Program is the assessments of pesticides in streams and groundwater in the United States, which are based on annual pesticide-use estimates. The details of the estimation of annual agricultural pesticide use for counties in the conterminous United States is described in detail elsewhere [39]. Briefly, for all States except California, pesticide use estimates are based on proprietary data on the amounts of pesticides applied to specific crops that is obtained from surveys of over 20,000 farm operations throughput the conterminous United States. The selection of the surveyed farm operations is based on the distribution of all such operations in the United States as enumerated by the United States Census of Agriculture, allowing for the estimation of total pesticide use by specific geographic regions (e.g., state, county). This information is combined with data from the United States Department of Agriculture on planted and harvested-crop acreage estimates to obtain national estimates for the use of specific pesticides within specific geographic areas (e.g., states, counties). Due to the sampling of farm operations for the collection of pesticide application data, some pesticide-by-crop combinations are not enumerated for some regions. As a result, the pesticide estimate for a given area is calculated using estimates from neighboring geographic regions; this is referred to as the EPest-high estimate and, consistent with prior studies, will be used in the current study [40]. Finally, for California, pesticide use estimates are based on data obtained from the California Department of Pesticide Regulation, which requires the reporting of all pesticides applied in the state. For the purposes of the current study, paraquat use estimates for each county in the conterminous United States for the period 1992 through 2017 were obtained from the NAWQA. A five-year moving average was calculated to account for annual fluctuations in paraquat use. For purposes of analysis, paraquat use was categorized by quintile of exposure: < 0.082, 0.082-0.321, 0.322-0.870, 0.871-2.282, and ≥ 2.282 kg/square mile.

Annual population counts by county, age, gender and race as well as county-level land area were obtained from the United States Bureau of the Census. In addition, percent of the population with a bachelor’s degree or higher was derived from the 2017 five-year American Community Survey, part of the Bureau of the Census; the county-level gross domestic product (GDP) of the agricultural industry was collected from data provided by the Bureau of Economic Analysis [41] for years 2001 through 2017; and 2013 urban and rural classifications were collected for each country from the National Center for Health Statistics [42]. For the GDP, the average of the years 2001-2017 was used in statistical analyses. For urban and rural classifications, counties were classified as either large central metros, large fringe metros, medium metros, small metros, micropolitan, or non-core counties.

### Statistical analysis

A dose-response curve was created by fitting a third-degree polynomial negative binomial regression line to the ESRD and paraquat exposure data. Separate curves were created for each lag period, and the curves were estimated up to 15 kg/sq. mile, which represents the 99th percentile of observed paraquat exposure. The association between the county-level paraquat incidence of ESRD per capita and the density of paraquat use was evaluated using rate ratios (RRs) and 95% confidence intervals (CIs) estimated from negative binominal regression models with and without adjustment for age, gender, race, percent of the population with a bachelor’s degree, agricultural GDP, and urban/rural classification. For purposes of statistical modeling, counts of ESRD cases and population were calculated for each combination of age, gender, and race prior to entering the data into the regression model. This was not done for the other covariates as they represent a single county-wide estimate.. Annual county-level paraquat density was calculated by dividing paraquat use estimates by county land area. For the associations between paraquat density (based on the five-year moving average) and ESRD incidence, paraquat density was lagged by 20, 15, 10, and 5 years to explore exposure latency. For 20-year lag models, ESRD incidence data prior to 2016 was excluded due to lack of 20-year lag paraquat use data (i.e., the first year of data with a full five-year moving average was 1996); for 15-year lag models, ESRD incidence data in 2010 was excluded for the same reason (Table 1). For all models, a test of linear trend across ordinal paraquat exposure quintile was performed by entering the exposure variable as a continuous variable into the models. *P*-values of ≤0.05 (two-sided) were considered statistically significant.

**Table 1:** Lagged exposure years for paraquat use based on end-stage renal disease (ESRD) diagnosis year

ESRD case year	Paraquat exposure (years included in the five-year moving average)			
	5-year lag	10-year lag	15-year lag	20-year lag
2010	2001-2005	1996-2000	1991-1995	1986-1990
2011	2002-2006	1997-2001	1992-1996	1987-1991
2012	2003-2007	1998-2002	1993-1997	1988-1992
2013	2004-2008	1999-2003	1994-1998	1989-1993
2014	2005-2009	2000-2004	1995-1999	1990-1994
2015	2006-2010	2001-2005	1996-2000	1991-1995
2016	2007-2011	2002-2006	1997-2001	1992-1996
2017	2008-2012	2003-2007	1998-2002	1993-1997
2018	2009-2013	2004-2008	1999-2003	1994-1998

## Results

**Fig. 1:**
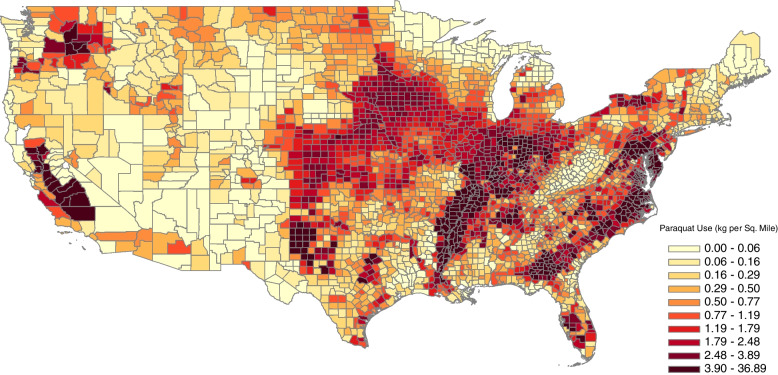
Average Estimated Annual Paraquat Application per Square Mile for 1992-2012 by County, United States

**Fig. 2:**
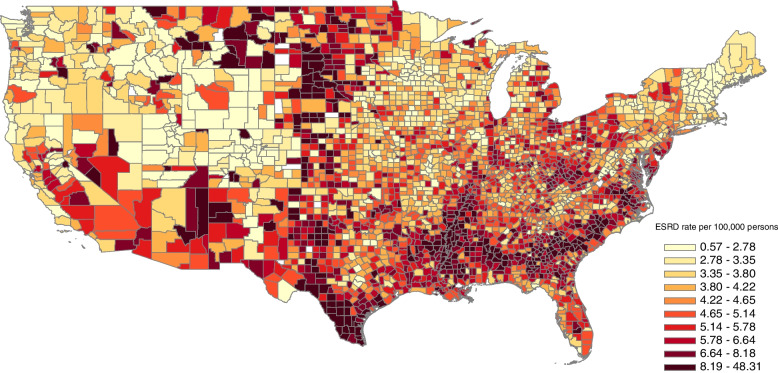
End-Stage Renal Disease (ESRD) Incidence per 10,000 Persons by County for 2010-2017, United States

Figure 1 demonstrates the density (i.e., average annual kilograms per square mile) of paraquat utilization across the conterminous United States from 1992 through 2012. From 1992 to 2012 the median annual amount of paraquat applied per county was similarly stable at approximately 350 kg, however, from 2013 through 2017 the median amount increased to approximately 550 kg. The annual total amount of paraquat applied per county also varied widely from a low of zero kilograms to a high of approximately 152,000 kg. Taking into account county land area, the median annual density of paraquat applied per county was 0.83 kg per square mile (min.: 0 kg per square mile; max: 88 kg per square mile). From 2010 through 2017, among 2974 counties in the contiguous United States (excluding the District of Columbia), the annual incidence rate of ESRD in the United States remained stable at approximately 5.4 per 10,000 population though county-level incidence rates varied widely from less than one to 125 per 10,000 population (excluding counties with populations of less than 500 people) with higher incidence rates observed in particular for counties within states in the southern United States including Texas, Mississippi, Alabama, Georgia, South Carolina, and North Carolina (Fig. 2).

The rate of ESRD increased with increasing paraquat use with noted increases for the 10-, 15-, and 20-year lagged models beginning between 25 and 30 kg/square mile exposure levels. Table 2 presents the RRs and 95% CIs for the association between county annual paraquat density and ESRD incidence. Compared to counties with the lowest density of paraquat application, the incidence rate of ESRD increased with increasing paraquat application density. The magnitude of the increase in ESRD rates generally increased with a decreasing lag in exposure. For example, considering a 20-year lag, those in the highest paraquat density quartile had a 21% higher rate of ESRD compared to the lowest quartile in crude regression models whereas for a 15-year lag the increase was 26%. In general, adjusted estimates were attenuated compared to the crude associations; however, the trend for increasing ESRD rate across increasing paraquat exposure quintile remained. The dose-response curves (Supplemental Fig. 1) for each lagged period replicated the observed relative associations with a nearly linear trend line through the 90th percentile of exposure (4 kg paraquat use/sq. mile) that was slightly stronger for the 10- and 15-year lagged periods. Beyond the 90th percentile exposure, the curves for the 10- and 20-year lags show a threshold response, the 15-year lag a decreasing trend in the rate (though still elevated to the no exposure level), and a continued rate increase for the 5-year lag; however, the observations beyond the 90th percentile are based on only a few counties and should be interpreted with caution.

**Table 2:** Crude^a^ and Adjusted^b^ Rate Ratios (RRs) and 95% Confidence Intervals (CIs) for the Association between Paraquat Density and ESRD Incidence Based on 20-Year, 15-Year, 10-Year and 5-Year Exposure Lags

	20 Year (2016-2018 cases)		15 Year (2011-2018 cases)		10 Year (2010-2018 cases)		5 Year (2010-2018 cases)	
	Crude RR (95% CI)	Adjusted RR (95% CI)	Crude RR (95% CI)	Adjusted RR (95% CI)	Crude RR (95% CI)	Adjusted RR (95% CI)	Crude RR (95% CI)	Adjusted RR (95% CI)
**Kilograms of Paraquat per Square Mile**								
< 0.082	1.00	1.00	1.00	1.00	1.00	1.00	1.00	1.00
0.082 to 0.321	0.99 (0.95-1.03)	0.99 (0.97-1.02)	1.02 (0.99-1.05)	1.01 (1.00-1.03)	1.02 (0.99-1.05)	1.02 (1.01-1.04)	1.03 (1.01-1.05)	1.03 (1.01-1.04)
0.322 to 0.870	1.11 (1.07-1.15)	1.05 (1.02-1.08)	1.10 (1.07-1.12)	1.05 (1.04-1.07)	1.07 (1.05-1.10)	1.06 (1.05-1.08)	1.07 (1.05-1.09)	1.05 (1.04-1.07)
0.871 to 2.282	1.09 (1.05-1.13)	1.06 (1.03-1.08)	1.10 (1.08-1.13)	1.08 (1.06-1.09)	1.10 (1.08-1.12)	1.09 (1.07-1.10)	1.09 (1.07-1.12)	1.07 (1.06-1.08)
> 2.282	1.21 (1.17-1.26)	1.07 (1.04-1.10)	1.26 (1.23-1.30)	1.10 (1.09-1.12)	1.22 (1.19-1.25)	1.11 (1.09-1.12)	1.21 (1.19-1.24)	1.07 (1.06-1.09)
P for trend	< 0.001	< 0.0001	< 0.001	< 0.001	< 0.001	< 0.001	< 0.001	< 0.001

^a^Estimated from a negative binomial regression with the natural log of the population as the model offset

^b^Adjusted for age group (< 50, ≥ 50 years), race, sex, percent of the county population with a bachelor’s degree or higher, county-specific gross domestic product of the agricultural industry, and urban/rural classification

## Discussion

According to the U.S. EPA paraquat is highly toxic and its application is limited to non-residential areas and only by licensed applicators. Additionally, it has identified paraquat as a potential risk to mammals and other animals and applicators are required to manage spray drift in order to minimize wildlife exposure. However, following a review of the toxicology and epidemiology literature on the human health effects of paraquat exposure, the EPA concluded that no clear link exists for any condition including Parkinson’s disease and cancer. Among the well-documented toxic effects of acute exposures, typically via ingestion, are respiratory and kidney failure, which in turn lead to death in the majority of cases. Despite the connection between paraquat exposure and AKI, there is little research regarding the effects of chronic paraquat exposure of renal disease. Using an ecological study design, the current study sought to assess the association between paraquat exposure and the incidence of ESRD in the United States. The results indicated a positive association between the density of paraquat utilization and the incidence of ESRD; this association persisted following adjustment for age, gender and race. Further, the strength of the association increased with increasing exposure density. To fully understand the implications of these results, it is useful to consider them within the broader context of research on paraquat exposure and kidney disease and the extent to which it supports a causal association.

These findings are consistent with the results of prior epidemiologic studies, including two from the Agricultural Health Study (AHS) that reported positive associations for end-stage renal disease (ESRD) [33, 34]. An AHS investigation of 55,580 male licensed pesticide applicators reported a statistically significant positive exposure-response trend for cumulative exposure to paraquat and ESRD [34]. A second AHS investigation among the wives of licensed pesticide applicators investigated the association between ESRD incidence and their husbands’ cumulative lifetime use of paraquat [33]. Relative to unexposed wives, those with any indirect exposure had a statistically significant two-fold increased risk of ESRD. Subgroup analysis of low and high exposure groups suggested a positive exposure response trend that failed to reach statistical significance. Nonetheless, there was only a small number of ESRD cases in each study, which limited the ability to access the exposure-response relationship. Jayasumana et al. compared cases of chronic kidney disease of unknown etiology (CKDu) to unaffected controls with respect to drinking water and occupational exposures in a region of Sri Lanka where CKDu is endemic [35]. Males, those engaged in farming, specifically pesticide application and consuming contaminated well water were among the significant risk factors identified; specifically, the ever use of paraquat, which was associated with a 2.5-fold increased odds. The authors hypothesize that well water contamination by heavy metals and pesticides contributes to the endemicity of CKDu in the region but that exposure to pesticides such as paraquat and, following its ban in 2010, glyphosphate, partly explain the higher incidence in male farmers. In a related study, Abdul et al. assessed urinary glyphosphate and paraquat levels and their association with markers of renal damage among rural farmers in CKDu endemic and non-endemic regions of Sri Lanka [36]. Urinary glyphosphate and paraquat levels and renal injury biomarkers were higher in CKDu endemic areas; however, associations were observed for glyphosphate but not paraquat.

The magnitude of the association between ESRD and paraquat exposure in the current study was small; however, this is not unexpected given the ecological nature of the study design. Even given the presence of a true association, the use of aggregate data may underestimate its magnitude due to a variety of factors. For example, exposure misclassification can occur if the county in which patients reside at the time of diagnosis is not the same as that of their etiologically relevant exposure period, which may itself be unknown. As described above, there have been three epidemiologic studies on this topic, all of which used individual rather than aggregate data, as was the case for the current study. The strength of the associations from those prior studies suggest that ESRD is at least twice as common among paraquat exposed versus unexposed individuals.

Among the features of an exposure-disease relationship that is often considered when evaluating causality is whether the relationship is specific; that is, the association exists among specific groups of people or is limited to a particular disease. As previously described, paraquat poisoning is known to induce AKI and though the mechanism of action underlying ESRD has not been investigated, that both acute and chronic kidney disease are implicated is telling. However, paraquat has been investigated for its connection to a variety of health outcomes, though it has only consistently been linked to Parkinson’s disease [8–17]. This does not per se remove the possibility that paraquat causes ESRD as one-to-one exposure-disease relationships are rare.

The use of ESRD incidence data from the USRDS permitted the current study to explore the issue of temporality. Nearly all of the research on paraquat exposure and kidney damage is based on acute ingestions, the timeline for which is short. The etiologically relevant period during which long-term, sub-toxic paraquat exposure leads to ESRD is unknown. The results of the current study suggest that a lag of between 5- and 15-years produced a stronger association than a 20-year lag, though the magnitude of the difference was small. Two of the three prior epidemiologic studies on this topic were based on the AHS, a prospective cohort study, and thus positive associations observed in those studies are bolstered by the temporal relationship between the exposures and outcomes afforded by the use of a prospective cohort study design.

The results of the current study revealed that the incidence of ESRD increased with increasing density of paraquat exposure; this was true regardless of the duration of the lag. Both of the prior studies from the AHS also evaluated the presence of a dose-response relationship. In the investigation of the wives of licensed pesticide applicators, the HR for those whose husbands’ were in the lowest category of cumulative lifetime use of paraquat was 1.36 and 1.78 for those in the upper category [33]. Despite the pattern of the HRs, the *p*-value for the trend was not significant, owing to the small numbers of ESRD cases in each of the exposure groups. A similar, though statistically significant, dose-response relationship was observed for the pesticide applicators themselves [34].

Though beyond the scope of this study, whether the association between paraquat exposure and ESRD is biologically plausible and coherent is an important consideration. Paraquat poisoning is a significant problem in certain parts of the world and, as such, the understanding of the associated health outcomes and the mechanisms underlying them is robust. Paraquat accumulates inside renal tubular cells resulting in reduction-oxidation cycling and an increase in reactive oxygen species [31, 32]. This damage reduces the kidney’s ability to eliminate paraquat, further aggravating the toxic effects within the body. Among the biomarkers associated with mortality following paraquat poisoning is rapid increase in serum creatinine, the increase in which appears to be dose dependent and occurs in response to oxidative stress [31, 32].

The results of the current study should be interpreted in light of a number of strengths and limitations. The USRDS provides a near complete enumeration of ESRD in the United States with a reported completeness rate of approximately 80 to 90% [43]. Given this high level of completeness, the opportunity for bias is minimal. Given the ecological nature of the study, the data regarding paraquat exposure was at the county rather than individual level and thus it is not possible to know whether associations at the population level also exist at the individual level. However, as discussed above, the existence of positive associations from other non-ecologic studies lends credence to the results of the current study. Additionally, the NAWQA Program estimates pesticide use using information obtained from surveys of farm operations; the estimates are not based on environmental sampling. However, studies have suggested that such environmental sampling may not reflect human exposure patterns due to the fate of pesticides once introduced into the environment [44]. Additional misclassification of paraquat exposure could have occurred due to migration as individuals were assigned paraquat levels based upon 5- through 20-year exposure lags. This may explain the weaker associations observed in the current study compared to the results of those of based on non-ecologic study designs. Though ecologic studies are primarily considered hypothesis generating, their use for the investigation of the health effects of pesticide exposure is particularly advantageous. This is due to the fact that individuals may not be able to accurately report pesticide exposures beyond their personal or occupational exposures. Exposure from drift during application as well as contaminated water sources would be impossible for individuals to report. As a final limitation, the 20-year lag for the current analysis was limited to a smaller subset of the 2010-2018 ESRD dataset; however, as the incidence of ESRD did not appreciably change during this time period, there is no reason to suspect a differential bias from the exclusion of 2010-2015 data, and the observed associations are likely underestimates of the true association for the 20-year lag analysis.

Consistent with prior research, the results of the current study indicate that community levels of paraquat use are positively associated with the incidence of ESRD. Though the mechanism underlying this association are not fully understood, the toxic effects of paraquat exposure on the kidney are well documented. Though the ban on paraquat use in many counties was motivated by its connection to Parkinson’s disease, evidence for its relationship to other health conditions such as ESRD mounts should bolster efforts to similarly ban its use in the United States.

## Supplementary Information

**Additional file 1: Supplemental Fig. 1.** Dose-Response Graph of Paraquat Use (Kg Used per Square Mile Land Area) and End-Stage Renal Disease Incidence (per 100,000 Persons) by lagged year model.

## Data Availability

Paraquat use data are publicly available from the United States Geologic Survey at https://water.usgs.gov/nawqa/pnsp/usage/maps/county-level/ ESRD incidence data are publicly available from the United States Renal Data System at https://usrds.org/data-query-tools/
